# Prenatal coparenting and attachment style in Japanese pregnant women: A cross-sectional survey

**DOI:** 10.1371/journal.pone.0309212

**Published:** 2024-08-29

**Authors:** Yui Masui, Akemi Yamazaki

**Affiliations:** 1 Department of Pediatric and Family Nursing, Division of Health Sciences, Graduate School of Medicine, Osaka University, Suita, Osaka, Japan; 2 Department of Maternity Outpatient, Osaka Women’s and Children’s Hospital, Izumi, Osaka, Japan; Universita Cattolica del Sacro Cuore Sede di Roma, ITALY

## Abstract

Developing prenatal coparenting is important for preparing couples for parenting immediately after childbirth, but knowledge of prenatal coparenting remains limited. Adult attachment style has been shown to be one of the factors during pregnancy that predict coparenting after childbirth, as well as a significant factor in the developmental process of the coparenting relationship. The present study mainly examines the relationship between prenatal coparenting as perceived by pregnant women and their attachment style. A cross-sectional survey was conducted at a tertiary emergency medical facility in Japan. Data from 181 pregnant women at 22–36 weeks’ gestation who completed a self-reported questionnaire consisting of the Prenatal Coparenting Scale (PCS), relationship-specific attachment styles, and characteristics were subjected to analysis. The mean age of the women in this study was 33.1 years (standard deviation = 5.2), 80 (44.2%) were expecting their first child, and 101 (55.8%) were expecting their second or subsequent child. Women’s attachment avoidance toward their mother (*r* = –.26), father (*r* = –.23), and partner (*r* = –.60) and attachment anxiety toward their partner (*r* = –.33) were significantly negatively correlated with PCS scores. When classified into two groups by fetal birth order, attachment avoidance and attachment anxiety toward the partner were significantly negatively correlated with PCS scores, regardless of fetal birth order. Unlike attachment style toward the partner, attachment avoidance toward the mother (*r* = –.33) and father (*r* = –.32) was significantly negatively correlated with PCS scores in the group of women expecting their second or subsequent child only. These results provide valuable insights into the relationship between prenatal coparenting and adult attachment style and deepen the understanding of prenatal coparenting. Future studies using longitudinal surveys and multivariate analyses could present relevant suggestions for specific types of support that promote the development of prenatal coparenting.

## Introduction

The transition to parenthood is a major life event that involves psychological and behavioral reorganizations and changes in dyadic relationships such as existing couple relationships [1]. Expectant couples who add a new parental role to their identity require joint work regarding how they will rear their children and establish a family [2].

Coparenting is a relationship focused on parenting [3, 4], described as an action shared by parents responsible for the care and rearing of their children [5]. Though much of the research on coparenting has accumulated findings from longitudinal studies of family and child adjustment after the birth of an infant, examining coparenting during pregnancy is considered a relatively new area of research [6]. Because it has been reported that coparenting develops at the behavioral level, even though actual child-rearing does not begin before the birth of an infant [7], it is important to develop coparenting during pregnancy in preparation for parenting immediately after childbirth. At the same time, measuring the quality of prenatal coparenting is essential in the process of promoting its development [8].

When assessing the coparenting behavior of pregnant couples, the Prenatal Lausanne Trilogue Play (PLTP) [9], a behavioral observation method that measures prenatal coparenting alliance, has mainly been used [10]. Specifically, the PLTP evaluates the interaction between expectant couples as they imagine the scene of meeting their baby for the first time and role-play together using a baby doll [9]. Recently, self-reported scales such as the Coparenting Relationship Scale-Father’s Prenatal Version (CRS-FPV) [11] for pregnant partners and the Prenatal Coparenting Scale (PCS) [12] for expectant women have also been developed. The CRS-FPV, a modified version of the Coparenting Relationship Scale (CRS) [13] that measures postnatal coparenting, assesses coparenting mental representations during pregnancy [11]. The PCS was developed with reference to Feinberg’s conceptual framework [5] for coparenting, which presents four constructs of coparenting: Childrearing agreement, Division of labor, Support-undermining, and Joint family management. The PCS focuses on couples’ interactions as parents of unborn babies.

Prebirth factors that predict coparenting in families with infant children have been reported to include some experiences in their families of origin [14, 15], personality [16–19], beliefs about parenting [15, 20, 21], expectations and concerns about future coparenting [6, 20, 21], expectations for a division of labor in child care [22], and quality of marital relationships [7, 23]. Adult attachment style, defined as a pattern of thoughts, feelings, and behaviors in a close relationship in adulthood [24, 25], is one of the personality traits included as a prenatal predictor. Individual differences in attachment style are often viewed in two dimensions: avoidance and anxiety [24, 26]. Attachment avoidance indicates the extent to which a person feels uncomfortable opening up to and relying on others, while attachment anxiety indicates the extent to which a person tends to worry about attachment-related concerns [27]. According to Bowlby’s attachment theory, becoming a parent is a chronic stressor and life event that activates the attachment system [28]. Attachment avoidance is characterized by a tendency to deactivate the attachment system, whereas attachment anxiety is characterized by a tendency to hyperactivate the attachment system [29]. Low scores for both attachment avoidance and attachment anxiety indicate secure attachment [26, 30]. The relationship between adult attachment style during pregnancy and coparenting after childbirth has been reported as follows. One study found that coparenting conflict was greater in postpartum families with mothers who were rated as having insecure attachment during pregnancy [18]. Pinto et al. [16] investigated the association between paternal attachment and coparenting representations from early pregnancy to 6 months postpartum and found that higher attachment avoidance in early pregnancy was associated with greater lack of coparenting support not only in early pregnancy, but also over time, and with greater coparenting conflict over time. Another study that examined the association between the attachment style of pregnant couples and maternal gatekeeping controlling father involvement in parenting reported that both maternal and paternal attachment styles are involved in the formation of maternal gatekeeping, respectively [31]. This finding on maternal gatekeeping, one of the components of the coparenting relationship [5], highlighted the importance of attachment style in the process of establishing a coparenting relationship [31]. Moreover, one review article [30] described that insecure attachment is negatively associated with parental characteristics and behaviors related to parenting. The above findings and results of the review suggest that a negative relationship may exist between the insecure attachment styles of pregnant women and prenatal coparenting. However, the relationship between the attachment style reported by pregnant women and prenatal coparenting has not yet been examined. Therefore, with the aim of deepening our understanding of families in the process of developing prenatal coparenting, this study aimed to investigate the relationship between the attachment style of pregnant women and prenatal coparenting. We also aimed to examine the relationship between prenatal coparenting and prenatal marital quality, which is strongly associated with perceptions of postnatal coparenting, and the characteristics of pregnant women. Couples expecting a second or subsequent child face the challenge of coordinating [32] and developing not only coparenting for the child, but also coparenting focused on the older child [33, 34]. In other words, the factors that affect prenatal coparenting to capture the parental interactions for the fetus may differ between couples expecting their first child and those expecting their second or subsequent child. Thus, the present study also examined cross-sectionally the relationship between variables in groups in which participants were divided into women expecting their first child and women expecting their second or subsequent child.

Given this background, the purpose of this study was, first, to examine the relationships between prenatal coparenting and the characteristics of pregnant women, marital satisfaction, and attachment style and second, to examine these relationships in terms of fetal birth order.

## Methods

### Design

The present study had a cross-sectional design.

### Procedure

This study, which involved pregnant women receiving antenatal care at a tertiary emergency medical facility in Japan, was conducted from November 26 to December 14, 2021. Pregnant women who met the inclusion criteria were selected by midwives or nurses belonging to the collaborating facility. When the selected women came to the hospital, the midwives and others obtained their verbal permission, and then the researcher explained the study both orally and in writing. Questionnaires were distributed and then collected either by posting them in a box placed in the outpatient reception area or by mail.

### Participants

The inclusion criteria were: (1) pregnant women who were 22 weeks or later and less than 37 weeks’ gestation, and (2) living with a partner. The exclusion criteria were: (1) multiple pregnancies, (2) a fetal abnormality, (3) a history of psychiatric complaints or a current diagnosis of a psychiatric disorder or obstetrician’s decision that psychosomatic consultation is necessary, (4) severe illness beyond the range of outpatient care, (5) cannot read or write Japanese, and (6) under 20 years of age.

### Measures

Sociodemographic characteristics, gestational age, fetal birth order, feelings when finding out about their pregnancy, obstetric complications, fetal anomalies, medical history, medical history of psychiatric complaints, and status of women’s return to their parents’ or in-laws’ home for childbirth were assessed using a self-report questionnaire. Referring to a previous study [35], obstetric complications in this study were considered to include gestational diabetes mellitus, hypertensive disorders of pregnancy, threatened premature delivery, and placental displacement.

Marital satisfaction was assessed using the 3-item Kansas Marital Satisfaction Scale (KMSS) developed by Schumm et al. [36] and translated by Sugawara and Takuma [37]. The KMSS correlates relatively strongly with the Revised Dyadic Adjustment Scale, which measures marital quality [38], and strong reliability was reported in a reliability generalization meta-analysis by Graham et al. [39]. The KMSS is evaluated on a 7-point Likert scale ranging from 1 (very dissatisfied) to 7 (very satisfied), with scores ranging from 3 to 21. Higher scores indicate higher marital satisfaction. Cronbach’s alpha was *α* = .96.

#### Attachment style

Pregnant women’s attachment styles were assessed using the Japanese version of Experience in Close Relationship-Relationship Structure (ECR-RS) [27, 40]. Komura et al. [40] considered validity as a single integrated concept, and verified the validity of this scale in an online panel survey based on evidence from internal structures such as factor structure and internal consistency, as well as from relationships with external variables such as depression and self-esteem. This scale consists of six avoidance items and three anxiety items, and it is rated on a 7-point Likert scale ranging from 1 (strongly disagree) to 7 (strongly agree). The total score is calculated after reversing the scores for the reversal items, taking a score range of 6–42 for avoidance and 3–21 for anxiety, with higher scores indicating more avoidant/anxious tendencies. The scale is designed so that respondents answer the same nine items for each of four intimate relationships (mother, father, partner, and friend); in this study, avoidance and anxiety toward the mother, father, and partner, respectively, were measured. Cronbach’s alpha for the Avoidance scale was *α* = .91 for the mother, *α* = .89 for the father, and *α* = .88 for the partner. Cronbach’s alpha for the Anxiety scale was *α* = .83 for the mother, *α* = .93 for the father, and *α* = .87 for the partner.

#### Prenatal coparenting

Prenatal coparenting was assessed using the 26-item PCS [12], which consists of two subscales: the Awareness of mutual support scale, and the Sharing of parenting that begins prenatally scale (S1 Table). The PCS is composed of 13 items, including “We think of how we can help each other in each situation” on the Awareness of mutual support scale, and 13 items, including “We are discussing how we would like to share household chores and childcare responsibilities after the birth of our child” on the Sharing of parenting that begins prenatally scale. Responses are rated on a 4-point Likert scale ranging from 1 (never) to 4 (always). The total score is calculated after reversing the score for one reversal item, taking a score range of 26–104, with higher scores indicating more coparenting during pregnancy. The PCS items were designed to capture the couple’s interactions through the pregnant woman. The scale development process is shown in S1 Fig. Internal consistency, retest reliability, and criterion-related validity were tested in Japanese pregnant women at 22 to 36 weeks’ gestation; detailed reports on scale development and psychometric evaluation are presented elsewhere [41]. The sample characteristics at the time of the psychometric evaluation [41] are shown in S2 Table.

In the present study sample, Cronbach’s alpha coefficients were *α* = .96 for the PCS, *α* = .92 for the Awareness of mutual support scale, and *α* = .93 for the Sharing of parenting that begins prenatally scale.

### Statistical analysis

The characteristics of pregnant women, KMSS, ECR-RS, and PCS scores were analyzed using descriptive statistics. Continuous variables were presented as means and standard deviations (*SD*s) or medians and interquartile ranges (IQRs). Categorical variables were presented as frequency (n) and proportion (%). Fetal birth order, occupational status, evaluation of financial situation, and education level were combined into binary variables. Specifically, fetal birth order was classified as “First child” or “Second or subsequent child”. Occupational status was classified as “Employed” or “Unemployed”. Evaluation of financial situation classified “Very good” and “Good” into “Good”, and “Fair” and “Poor” into “Poor”. For education level, “College” and “Graduate school” were grouped into “≥ College graduate” and the remainder were grouped into “< College graduate”. The Mann–Whitney *U* test was used to detect differences between PCS scores and binary variables (characteristics such as occupational status). Spearman’s rank correlation coefficient was used for the associations between PCS scores and continuous variables (characteristics such as age, KMSS, ECR-RS). Next, the participants were divided into two groups, women expecting their first child and women expecting their second or subsequent child, and the same analyses as for all participants were performed for each group. All participants were married, except for three who were engaged, so variables related to marital status were not included in subsequent analyses. All analyses in the present study were performed using IBM SPSS Statistics Ver. 28.0 (IBM Japan Tokyo, Japan), with a significance level of 5%.

### Ethical considerations

This study was approved by Ethics Review Board of Osaka University Hospital (approval No.: 21021–2) and Osaka Women’s and Children’s Hospital Ethics Committee (approval No.: 1444–2). The researcher explained the purpose and methods of the study, the voluntary nature of participation, privacy protection, data management, and the fact that consent could not be withdrawn after the questionnaires were submitted because the collected questionnaires were numbered and handled to all participants before the study began. All participants were assured that they would not be disadvantaged in any way by their cooperation in the study. The cover page of the questionnaire form stated “Please indicate your intention to cooperate with this survey” and included a box that could be checked “Agree” or “Disagree”. When the questionnaires were collected with both the “Agree” checkbox and the date of their response the survey was completed, consent to cooperate in the study was considered obtained.

## Results

The questionnaire was distributed to 230 pregnant women, among whom, responses were received from 228. From the returned questionnaires, 47 were excluded from the analysis: 20 who met the exclusion criteria, 9 who had missing PCS or KMS values, 9 who were dishonest in their responses, 8 who did not provide written consent, and 1 who had missing values for 10% or more of the attribute items. Thus, 181 women (79.4% response rate) were included in the final analysis. Tables 1 and 2 show the distribution of the participants’ characteristics and scores for each scale, respectively. The mean age was 33.1 (*SD* = 5.2) years, 55 women (30.4%) were in the second trimester, and 126 (69.6%) were in the third trimester. Furthermore, 80 women (44.2%) were expecting their first child, and 101 (55.8%) were expecting their second or subsequent child. More than half of the women (62.8%) had jobs, and 107 (59.8%) reported that they were “very good” or “good” regarding their financial situation. Finally, 89 women (49.4%) had a college degree or higher.

**Table 1:** Women’s characteristics.

Variable	Total (n = 181)		Fetal birth order			
			First (n = 80)		Second or subsequent (n = 101)	
	n	Mean or n	n	Mean or n	n	Mean or n
Characteristic						
Age (years) (*SD*)	181	33.1 (5.2)	80	32.4 (5.5)	101	33.6 (4.8)
Family structure						
Nuclear family (%)	181	169 (93.4)	80	75 (93.8)	101	94 (93.1)
Other (%)	181	12 (6.6)	80	5 (6.3)	101	7 (6.9)
Gestational age (weeks) (*SD*)	181	29.8 (4.5)	80	30.0 (4.4)	101	29.6 (4.6)
Fetal birth order						
First (%)	181	80 (44.2)				
Second (%)	181	62 (34.3)				
Third (%)	181	27 (14.9)				
Fourth and above (%)	181	12 (6.6)				
Feelings when I found out about my pregnancy						
Happy (%)	181	160 (88.4)	80	72 (90.0)	101	88 (87.1)
Other (%)	181	21 (11.6)	80	8 (10.0)	101	13 (12.9)
Pregnancy complications						
Yes (%)	181	32 (17.7)	80	13 (16.3)	101	19 (18.8)
No (%)	181	149 (82.3)	80	67 (83.8)	101	82 (81.2)
Past medical history						
Yes (%)	181	34 (18.8)	80	18 (22.5)	101	16 (15.8)
No (%)	181	147 (81.2)	80	62 (77.5)	101	85 (84.2)
Occupational status						
Full-time job (%)	180	47 (26.1)	80	32 (40.0)	100	15 (15.0)
Part-time job (%)	180	13 (7.2)	80	4 (5.0)	100	9 (9.0)
Maternity leave/leave of absence (%)	180	53 (29.4)	80	25 (31.3)	100	28 (28.0)
Homemaker/student (%)	180	67 (37.2)	80	19 (23.8)	100	48 (48.0)
Evaluation of financial situation						
Very good (%)	179	2 (1.1)	79	0 (0)	100	2 (2.0)
Good (%)	179	105 (58.7)	79	51 (64.6)	100	54 (54.0)
Fair (%)	179	63 (35.2)	79	25 (31.6)	100	38 (38.0)
Poor (%)	179	9 (5.0)	79	3 (3.8)	100	6 (6.0)
Education level						
Junior high school (%)	180	9 (5.0)	80	5 (6.3)	100	4 (4.0)
High school (%)	180	29 (16.1)	80	9 (11.3)	100	20 (20.0)
Junior college/technical school (%)	180	53 (29.4)	80	23 (28.7)	100	30 (30.0)
College (%)	180	86 (47.8)	80	41 (51.2)	100	45 (45.0)
Graduate school (%)	180	3 (1.7)	80	2 (2.5)	100	1 (1.0)
Currently returned to parents’ home for delivery						
Yes (%)	181	23 (12.7)	80	13 (16.3)	101	10 (9.9)
No (%)	181	158 (87.3)	80	67 (83.8)	101	91 (90.1)

The total and subscale scores for all measures used in this study were not normally distributed. The median (IQR) KMSS score for all women was 18.0 (17.0–21.0). The median (IQR) ECR-RS scores in the three domains for all women were: 11.0 (7.0–18.0) and 3.0 (3.0–3.0) for avoidance and anxiety toward the mother, respectively; 20.0 (12.0–27.0) and 3.0 (3.0–4.0) for the father, respectively; and 9.0 (6.0–13.0) and 3.0 (3.0–6.0) for the partner, respectively. The median (IQR) PCS score for all women was 80.0 (68.0–90.0).

**Table 2:** Median and interquartile range (IQR) of scores on the measures used in this study.

Measure	Total (n = 181)		Fetal birth order			
			First (n = 80)		Second or subsequent (n = 101)	
	n	Median (IQR)	n	Median (IQR)	n	Median (IQR)
KMSS scores	181	18.0 (17.0–21.0)	80	18.5 (18.0–21.0)	101	18.0 (15.0–20.5)
**Attachment style**						00
ECR-RS-mother avoidance	180	11.0 (7.0–18.0)	79	12.0 (7.0–18.0)	101	11.0 (7.0–18.5)
ECR-RS-mother anxiety	180	3.0 (3.0–3.0)	79	3.0 (3.0–4.0)	101	3.0 (3.0–3.0)
ECR-RS-father avoidance	174	20.0 (12.0–27.0)	76	21.0 (14.0–26.0)	98	19.5 (12.0–28.0)
ECR-RS-father anxiety	173	3.0 (3.0–4.0)	76	3.0 (3.0–3.0)	97	3.0 (3.0–4.0)
ECR-RS-partner avoidance	179	9.0 (6.0–13.0)	78	9.0 (6.0–12.0)	101	9.0 (6.0–13.5)
ECR-RS-partner anxiety	181	3.0 (3.0–6.0)	80	3.0 (3.0–5.0)	101	3.0 (3.0–6.0)
**Prenatal coparenting**						
PCS scores	181	80.0 (68.0–90.0)	80	80.5 (71.5–91.5)	101	77.0 (63.0–90.0)
Awareness of mutual support	181	42.0 (36.0–47.0)	80	44.0 (41.0–48.0)	101	41.0 (33.5–46.0)
Sharing of parenting that begins prenatally	181	37.0 (31.0–44.0)	80	38.0 (32.0–42.0)	101	37.0 (31.0–44.0)

### Relationships among the PCS, characteristics of pregnant women, and the ECR-RS

The binary variables that showed significant differences in PCS scores were evaluation of financial situation (*z* = –2.48, *p* = .01) and currently returned to parents’ or in-laws’ home for delivery (*z* = 2.03, *p* = .04) (Table 3). The scores were positively correlated with gestational age (*r* = .24, *p* < .001) and KMSS scores (*r* = .66, *p* < .001). On the other hand, PCS scores were negatively correlated with ECR-RS-mother avoidance (*r* = –.26, *p* < .001), ECR-RS-father avoidance (*r* = –.23, *p* = .003), ECR-RS-partner avoidance (*r* = –.60, *p* < .001), and ECR-RS-partner anxiety (*r* = –.33, *p* < .001) (Table 4). The results of the analyses of the two PCS subscale scores are presented in S3–S5 Tables.

**Table 3:** Comparison of PCS Scores by Women’s Characteristics (Binary Variables).

	PCS score											
	Total (n = 181)				Fetal birth order							
					First (n = 80)				Second or subsequent (n = 101)			
	n	Mean (*SD*)	*z*	*p*	n	Mean (*SD*)	*z*	*p*	n	Mean (*SD*)	*z*	*p*
Family structure												
Nuclear family	169	78.1 (15.9)	00.36	.720	75	80.4 (13.6)	01.05	.300	94	76.3 (17.4)	–0.25	.8100
Other	12	80.8 (10.6)			5	87.2 (9.5)			7	76.1 (9.2)		
Fetal birth order												
First child	80	80.8 (13.5)	–1.70	.090								
Second or subsequent child	101	76.3 (16.9)										
Feelings when I found out about my pregnancy												
Happy	160	78.9 (15.8)	–1.93	.050	72	81.4 (13.4)	–1.34	.180	88	76.8 (17.3)	–1.21	.2300
Other	21	73.5 (13.7)			8	74.9 (13.7)			13	72.7 (14.1)		
Pregnancy complications												
Yes	32	78.0 (17.8)	0.18	.860	13	83.0 (15.2)	00.52	.600	19	74.6 (19.1)	–0.26	.7900
No	149	78.3 (15.1)			67	80.3 (13.2)			82	76.6 (16.5)		
Past medical history												
Yes	34	75.9 (15.2)	–1.01	.310	18	77.3 (10.7)	–1.38	.170	16	74.2 (19.3)	–0.46	.6500
No	147	78.8 (15.7)			62	81.8 (14.1)			85	76.7 (16.5)		
Occupational status												
Employed	113	78.7 (14.2)	0.19	.840	61	79.7 (12.5)	–1.45	.150	52	77.5 (16.0)	00.64	.5200
Unemployed	67	77.4 (17.9)			19	84.2 (16.0)			48	74.7 (18.0)		
Evaluation of financial situation												
Good	107	80.8 (14.3)	–2.48	.01*	51	80.5 (14.4)	00.21	.840	56	81.1 (14.4)	–3.10	.002*
Poor	72	74.4 (16.9)			28	81.1 (12.2)			44	70.2 (18.2)		
Education level												
< College graduate	91	79.0 (14.7)	–0.69	.490	37	81.9 (11.3)	–0.78	.430	54	76.9 (16.4)	–0.50	.6200
≥College graduate	89	77.3 (16.5)			43	79.8 (15.2)			46	75.0 (17.4)		
Currently returned to parents’ home for delivery												
Yes	23	84.0 (13.7)	2.03	.04*	13	87.9 (13.1)	02.29	.02*	10	79.1 (13.5)	00.52	.6100
No	158	77.4 (15.7)			67	79.4 (13.2)			91	76.0 (17.3)		

*p < .05.

**Table 4:** Correlations among measures of prenatal coparenting, attachment style, and women’s characteristics (Continuous Variables).

	Total (n = 181)		Fetal birth order			
			First (n = 80)		Second or subsequent (n = 101)	
	n	*r*	n	*r*	n	*r*
**PCS**						
Age	181	–.16*	80	–.13	101	–.19
Gestational age	181	.24**	80	.25*	101	.25*
KMSS	181	.66**	80	.54**	101	.73**
ECR-RS-mother avoidance	180	–.26**	79	–.20	101	–.33**
ECR-RS-mother anxiety	180	–.15*	79	–.20	101	–.11
ECR-RS-father avoidance	174	–.23**	76	–.09	98	–.32**
ECR-RS-father anxiety	173	–.06	76	–.03	97	–.06
ECR-RS-partner avoidance	179	–.60**	78	–.51**	101	–.66**
ECR-RS-partner anxiety	181	–.33**	80	–.34**	101	–.33**

*p < .05.

**p < .01.

### Relationships among the PCS, characteristics of pregnant women, and the ECR-RS by fetal birth order

#### Women expecting their first child

A significant difference in PCS scores was found between women who had returned to their parents’ or in-laws’ home and those who had not (*z* = 2.29, *p* = .02) (Table 3). The scores were higher the closer the gestational age was to full-term (*r* = .25, *p* = .028) and the higher the KMSS score (*r* = .54, *p* < .001). The higher were ECR-RS-partner avoidance (*r* = –.51, *p* < .001) and ECR-RS-partner anxiety (*r* = –.34, *p* = .002) scores, the lower were the PCS scores (Table 4).

#### Women expecting their second or subsequent child

A significant difference in PCS scores was found between women in the “Good” and “Poor” groups for evaluation of financial situation (*z* = –3.10, *p* = .002) (Table 3). PCS scores were higher the closer the gestational age was to full-term (*r* = .25, *p* = .013) and the higher the KMSS score (*r* = .73, *p* < .001). The higher were ECR-RS-mother avoidance (*r* = –.33, *p* < .001), ECR-RS-father avoidance (*r* = –.32, *p* = .001), ECR-RS-partner avoidance (*r* = –.66, *p* < .001), and ECR-RS-partner anxiety (*r* = –.33, *p* < .001), the lower were the PCS scores (Table 4). The results of the analyses of the two PCS subscale scores by fetal birth order are presented in S3–S5 Tables.

## Discussion

The present study primarily examined the relationship between prenatal coparenting and adult attachment style. Furthermore, we also investigated the relationship between prenatal coparenting and pregnant women’s characteristics. The analyses were conducted not only for all participants, but also by group, where participants were divided into two groups: women expecting their first child and women expecting their second or subsequent child. The items that showed a relationship with PCS scores for the participants as a whole were: evaluation of financial situation, return to their parents’ or in-laws’ home for childbirth, gestational age, marital satisfaction, avoidance toward the mother, father, and partner, and anxiety toward the partner. The same trend was observed for gestational age, marital satisfaction, avoidance toward the partner, and anxiety, regardless of fetal birth order, but the results differed for evaluation of financial situation, return to their parents’ or in-laws’ home, and avoidance toward the mother and father. These results are discussed below.

### Relationships among the PCS, characteristics of pregnant women, and the ECR-RS

Regarding the evaluation of their financial situation, PCS scores were significantly higher for those who rated their financial situation as good. With a focus on paternity leave and relationship quality, Petts et al. [42] reported that the likelihood of paternity leave-taking depends on socio-economic status, and that paternity leave-taking predicts the quality of postnatal coparenting. For families under difficult socio-economic conditions, the findings of Petts et al. [42] suggested that easier access to paternity leave would encourage fathers to become more involved in child-rearing. Because an association between socio-economic status and coparenting after the birth of the child was identified in the conceptual framework of Feinberg [5], we believe that the results of the present study, which were obtained during pregnancy, are also valid. In addition, PCS scores were significantly higher with increases in gestational age. This result is understandable because, in general, couples in the later trimester have more opportunities to discuss child-rearing among themselves as they prepare to welcome their children. Future longitudinal studies conducted to capture various aspects of prenatal coparenting among pregnant women could be expected to provide additional insights. PCS scores were significantly higher for the respondents who were currently returning to their parents’ or in-laws’ home. In Japan, the custom of a postpartum support system called *satogaeri bunben* [43, 44], in which women return to their parents’ or in-laws’ homes around the time of childbirth, has been common and is still practiced today. Most women return to their parents’ homes from around 8 months’ pregnant to 6–8 weeks’ postpartum [44, 45], receiving support mainly from their own mothers [46]. Thus, it can be said that the women in the present study who responded that they were currently returning home were likely to be in the last trimester of pregnancy. Furthermore, the above result that PCS scores were higher with increases in gestational age cannot exclude the possibility that gestational age affected the results, and thus, further analysis adjusting for gestational age is needed. However, to our knowledge, the finding that women and their partners spending time apart before childbirth [47] may influence prenatal coparenting is new. PCS scores were significantly higher for higher marital satisfaction. Many prior studies have demonstrated an association between the couple’s relationship quality during pregnancy and coparenting after the birth of the child. Because both coparenting and couple relationships are relationships between couples [3, 5], they can be expected to be related even during the pregnancy period, and the results of this study support this relationship.

Many scales measuring adult attachment styles that focus on the lover or spouse have been used [40]. Additionally, much of the previous literature on the transition to parenthood has addressed partner-related attachment styles. To our knowledge, this is the first study to report the attachment styles of pregnant women to their mothers, fathers, and partners in the same paper using the ECR-RS [27], which allows measurement of relationship-specific attachment styles. Our literature search failed to reveal any studies using the ECR-RS in Japanese pregnant women, and these scores could not be compared in the present study. Hence, our search of the literature on the use of the ECR-RS among pregnant women identified a study in Portugal [48] that used it during the second trimester and a study in Australia [49] that used it during the third trimester. These reports imply that women in the third trimester may have higher average attachment avoidance and anxiety scores than women in the second trimester. Since approximately 70% of the participants in this study were pregnant women in the third trimester, the attachment avoidance and anxiety scores may have tended to be higher.

Contrary to some previous studies, this study showed a relationship between attachment style toward the partner during pregnancy and prenatal coparenting. Alves et al. [48] reported that higher avoidance toward the partner predicted lower common dyadic coping during the transition to parenthood, but found no such association with anxiety toward the partner. Since common dyadic coping [48], a skill in which couples cope together with common stressors, has been suggested to be a resource that promotes supportive coparenting [50], avoidance toward the partner and prenatal coparenting were considered to be negatively correlated. Alves et al. [48] also noted that the demonstration of the hypothesis that there is no significant association between common dyadic coping and anxiety toward the partner is a reflection of ambivalent attitudes toward partners expressed by anxious individuals who worry about rejection or abandonment [29]. However, their remarks did not explain our finding that anxiety toward the partner was significantly associated with prenatal coparenting, although the strength of the association was weak. The relationship between parenting and attachment style as measured by self-reporting is complex, with no consistent findings on relationships between each area of both [30], and the stability of attachment styles is lower in relationships with partners than in those with parents [51]. Given these insights, it is difficult to deepen the discussion about attachment anxiety toward partners based on our findings from the cross-sectional survey. Further research, including longitudinal studies of the relationship between attachment style and prenatal coparenting, is needed.

### Relationships among the PCS, characteristics of pregnant women, and the ECR-RS by fetal birth order

No significant differences in PCS scores by evaluation of financial situation were found among women expecting their first child. However, among women expecting their second or subsequent child, a significant difference in PCS scores was found between women in the “Good” and “Poor” financial situation groups. O’Laughlin and Anderson [52] reported that compared with a group of parents who wanted children in the future, a group of parents with one or more children expected that having children would be a longer-term financial burden. We consider that the findings show different results depending on fetal birth order because economic stress is more likely to be felt at the second or subsequent child than at the first, when actual childcare has not yet started. For women expecting their first child, PCS scores were significantly higher for those who returned to their parents’ or in-laws’ home, whereas for women expecting their second or subsequent child, the trend was the same as that for the first child, although not significantly so. In a previous study, women reported that the most common reason for choosing *satogaeri bunben* was anxiety about their first experience [44]; the same tendency was observed in the present study. On the other hand, the reasons for not returning home included the availability of support from the biological mother at home and taking care of the older child [44], and thus, it can be expected that the support system within and outside the family is likely to have an impact on whether a person returns home. However, this was not taken into account in the present study and remains an issue for future research.

The significant correlation between PCS scores and avoidance toward the mother and father among women expecting their second or subsequent child is discussed in terms of the presence of childcare for the older child. They are involved in coparenting, which focuses on each of the older children, as well as the child. Yan et al. [53] reported that the quality of coparenting in the origin family as perceived by couples during pregnancy predicted dyadic adjustment and negative interactions as perceived by parents and their partners after the birth of their first child. They also showed that this prediction was mediated by attachment representations to the partner. The quality of coparenting and parent–child attachment are related in couples engaged in child-rearing [54], and early parent–child attachment influences the attachment relationship with a future intimate object for that child [55]. Because the family is considered a system, dyadic and triadic relations within the family are interrelated, and thus, coparenting to the child and older child may also influence each other. In the case of women expecting their second or subsequent child, attachment style to the parents was related to coparenting to the older child, which was also correlated with PCS scores, whereas no correlation was found in the case of women expecting their first child.

### Strengths and limitations

This study has several study limitations. First, the large number of women in the last trimester of pregnancy and the fact that data collection was conducted at a single site limit the generalizability of the results. Second, because this was a cross-sectional study, we were unable to capture changes or trends in variables over the gestation period, which made it impossible to examine causal relationships between variables. Third, the PCS, which measures prenatal coparenting, is a scale that has been tested for reliability and validity in Japanese women; however, considering the influence of cultural backgrounds on events related to pregnancy, childbirth, and parenting, future examinations of the psychometric properties of the PCS are required for other racial and ethnic groups. Finally, the sample size in this study was insufficient for multivariate analysis.

Despite these limitations, we believe that the results of the present study provide useful suggestions regarding the relationship between prenatal coparenting and adult attachment style. Our findings focusing on prenatal coparenting, an area of increasing research attention, may therefore contribute important information for future studies.

## Conclusions

In the present study, higher attachment avoidance toward parents and partners as rated by pregnant women was associated with lower prenatal coparenting. In addition, women’s higher attachment anxiety toward their partners was associated with lower prenatal coparenting. We measured the attachment styles of pregnant women to their parents as well as their partners, and found that the relationship between prenatal coparenting and attachment style as perceived by pregnant women may differ depending on fetal birth order. These suggestions regarding the relationship between prenatal coparenting and attachment style have improved our understanding of families in the developmental process of prenatal coparenting. Longitudinal design studies and multivariate analyses will be needed in future studies to identify causal relationships between variables. Further research could lead to specific recommendations to support the development of prenatal coparenting.

## Supporting information

S1 Fig Process of Prenatal Coparenting Scale (PCS) development. (DOCX)

S1 Table List of 26 items in the PCS. (XLSX)

S2 Table Sample characteristics when the reliability and validity of 26 items in the PCS were tested. (XLSX)

S3 Table Comparison of Awareness of mutual support scores by women’s characteristics (binary variables). (XLSX)

S4 Table Comparison of Sharing of parenting that begins prenatally scores by women’s characteristics (binary variables). (XLSX)

S5 Table Correlations among measures of prenatal coparenting, attachment style, and women’s characteristics (continuous variables). (XLSX)

S1 Data Dataset. (XLSX)
