# Supplementary material for: Prenatal coparenting and attachment style in Japanese pregnant women: A cross-sectional survey
Source: PLoS One. 2024 Aug 29;19(8):e0309212. doi: 10.1371/journal.pone.0309212 (PMC11361575; doi:10.1371/journal.pone.0309212)
Supplement: S1 Fig — (DOCX) [file pone.0309212.s001.docx]

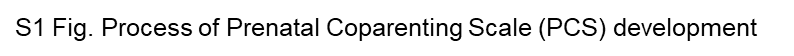

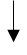

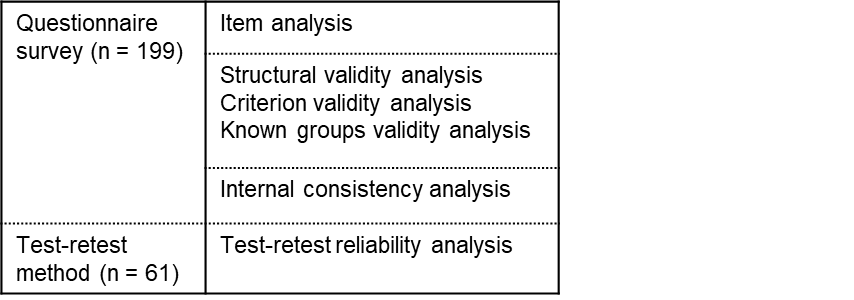

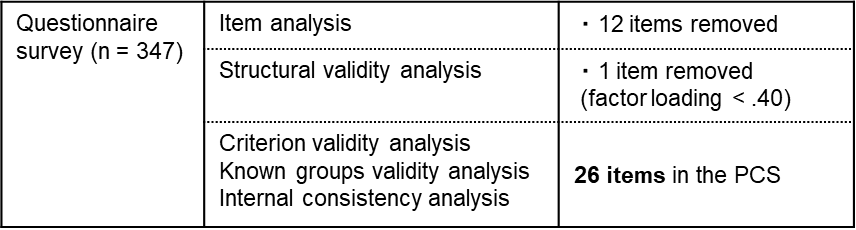

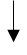

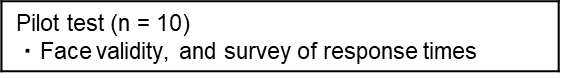

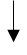

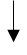

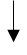

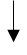

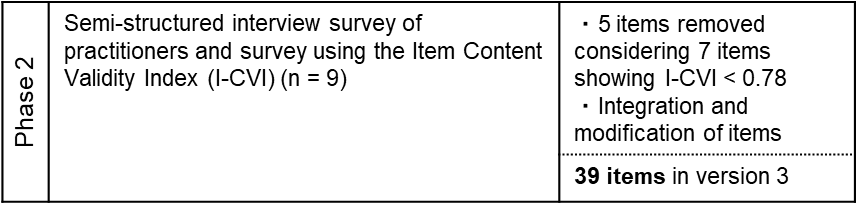

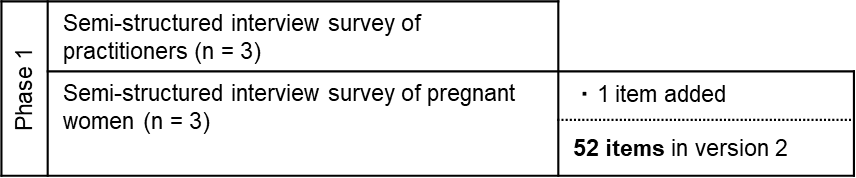

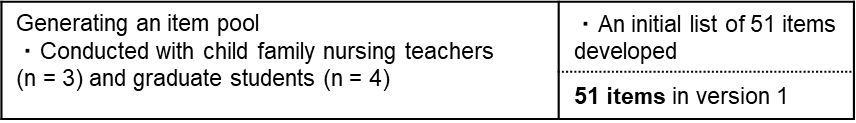

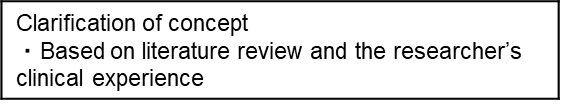


Examination of the reliability and validity of the 26 items in the PCS

Face and content validity of the draft scale/

Selecting the items

Development of
a draft scale
